# The influence of locus of control on self-rated health in context of chronic disease: a structural equation modeling approach in a cross sectional study

**DOI:** 10.1186/1471-2458-14-492

**Published:** 2014-05-23

**Authors:** Erik Berglund, Per Lytsy, Ragnar Westerling

**Affiliations:** 1 Department of Public Health and Caring Sciences, Uppsala University, Box 564, SE-751 22 Uppsala, Sweden

**Keywords:** Self-rated health, Locus of control, Chronic disease, Cardiovascular disease, Diseases burden, Logistic regressions, Path model, SEM

## Abstract

**Background:**

Self-rated health is a robust predictor of several health outcomes, such as functional ability, health care utilization, morbidity and mortality. The purpose of this study is to investigate and explore how health locus of control and disease burden relate to self-rated health among patients at risk for cardiovascular disease.

**Methods:**

In 2009, 414 Swedish patients who were using statins completed a questionnaire about their health, diseases and their views on the three-dimensional health locus of control scale. The scale determines which category of health locus of control – internal, chance or powerful others – a patient most identifies with. The data was analyzed using logistic regression and a structural equation modeling approach.

**Results:**

The analyses showed positive associations between internal health locus of control and self-rated health, and a negative association between health locus of control in chance and powerful others and self-rated health. High internal health locus of control was negatively associated with the cumulative burden of diseases, while health locus of control in chance and powerful others were positively associated with burden of diseases. In addition, age and education level had indirect associations with self-rated health through health locus of control.

**Conclusions:**

This study suggests that self-rated health is positively correlated with internal locus of control and negatively associated with high locus of control in chance and powerful others in patients at high risk for cardiovascular disease. Furthermore, disease burden seems to be negatively associated with self-rated health.

## Background

Self-rated health (SRH) is one of the most widely used measures of personal perceived health. SRH, when measured via one question, is a robust predictor of several health outcomes, such as functional ability [1,2], returning to work after coronary artery disease [3], health care utilization [4], morbidity [5,6], and mortality [7-11]. It has been shown that SRH is a more reliable predictor for future health and mental health than other more objective measures [12]. SRH is often used as an outcome measure in public health-based population surveys and health service interventions because of its predictable functions.

Little is known about the mechanism behind SRH. SRH is a complex predictor and early attempts to use SRH as only a proxy for disease burden have been unsuccessful [12]; even then SRH seems to be affected by objective health to a great extent [13]. Several factors have shown associations with SRH, among them low income [14], social isolation [14], work related factors [15], psychological and social factors [15]. Possible explanations for SRH’s functioning include SRH representing an individual’s general perception of health, including biological, psychological and social dimensions. Therefore SRH might be more sensitive in health monitoring than other objective or clinical measures of health [4]. However SRH is not considered to be easily affected by temporary situations [15] and despite this, earlier studies have found that some powerful predictors of SRH are potentially modifiable [16].

Locus of control (LoC), and health locus of control, were developed from social-learning theory and refer to the degree of control that people believe they possess over their personal health [17]. Health locus of control is, in the multidimensional form, attributed to internal factors, chance, or external factors [18]. People with high internal LoC believe that their personal health-related outcomes are mostly determined by their own choices and actions. People with a high LoC in chance believe that their health outcomes are mostly determined by luck or chance. People with a high external LoC believe that other powerful people, such as health care providers, will determine their health outcome. LoC is considered to be quite stable over time, and the original construct was defined as a quite stable personality trait [19]. This has been further supported by clinical data [20]. Nevertheless, research also indicates that although one’s overall LoC may be stable across time, changes can be seen in interventions targeting these factors with cognitive training [21]. In addition, general health education based on information, face-to-face meetings and healthy lifestyle training seems to have an impact on control feelings [22]. LoC has been shown to have an impact on risk factors, health and diseases. Gale et al. found that high internal LoC at age 10 is protective against obesity, being overweight, poor self-rated health and psychological distress at the age of 30 [23]. High internal LoC also reduced the risk of high blood pressure among women [23]. Lack of internal LoC has been associated with being overweight/obese when compared with normal weight women [24]. There are some results that indicate that LoC is associated with chronic diseases [25-27], SRH [28,29], and mortality [30,31]. LoC beliefs have been associated with successful treatment outcomes; those patients with stronger internal beliefs had gained more from the treatment [32]. Associations between LoC and treatment adherence behavior have been found in earlier studies [33-35]. LoC as a single factor plays a modest role in explaining health behavior [36]. In some studies LoC is considered to be a mediating variable [37,38]. However empirical studies are inconsistent in their findings and some studies do not find any association or little explanatory power between LOC and health behavior [39,40].

Living life with a chronic disease faced with long-term treatment can influence perceived health. Today, cardiovascular disease (CVD) is the most common disease and the leading cause of death in the industrialized world [41,42]. To reduce the risk for CVD, one must maintain low cholesterol levels. Statins, cholesterol lowering drugs, are therefore one of the most common long-term treatments. A not fully explored and interesting consideration is how LoC relates to SRH and diseases in a population with risk for CVD.

This study aims to investigate how health LoC and disease burden relates to SRH among patients at risk for CVD, and to explore a framework to examine how different factors are related to each other and SRH.

## Methods

### Sample

A cross-sectional design was used for this study. A total of 600 questionnaires were distributed in May 2009 to the 28 operating pharmacies within the county of Uppsala in central Sweden. The number of questionnaires distributed to each pharmacy was proportional to the number of statin prescription sales. The employees of each pharmacy were instructed to approach every patient who came in to obtain their statin prescription. There were no inclusion criteria other than the statin prescription requisite, and no exclusion criteria. After receiving oral and written information about the study by the pharmacist, patients who agreed to participate were handed a questionnaire to take home and complete, and then return by post. The number of patients declining to participate was registered for control of non-participants. The first page of the questionnaire contained precise information on the purpose of the study. Completed questionnaires were returned anonymously in a prepaid envelope. All questionnaires returned within three months were included in the study. A total of 697 statin users were asked to participate, 109 people declined participation and 588 questionnaires were handed out. One pharmacy failed to distribute their questionnaires. Questionnaires were returned by 414 individuals with a response rate for the distributed questionnaires as 70.4% (414/588) and the overall response rate 59.4% (414/697). The study population consisted of slightly more men (51.0%) than women (49.0%). This dataset has been used in a previous study [35].

### Measures

The questionnaire contained a total of 76 questions. The main data types and measures included were:

Demographic data were collected using questions that assessed the respondent’s gender, age and educational level (categorized as compulsory school, secondary school or equivalent, or university).

Wallston’s Multidimensional Health Locus of Control scale (MHLC) was used to determine health locus of control [18]. Respondents used a Likert scale to rate their agreement with six statements that characterized each LoC dimension: internal (I), chance (C) and powerful others (PO). Each dimension had a possible range of scores from 6 to 36 per scale. Wallston’s MHLC scale is commonly used in patients with chronic diseases [43-45]. The MHLC scale is considered to be valid [36], and the scale has been tested in different countries [46].

Information about the patient’s diseases was collected through the question: Do you have any chronic diseases, illnesses or disability or any incapability due to accident? Respondents stating “yes” were asked to specify what kind of illness using a list of 14 common health problems, including: Allergy, diabetes, symptoms of CVD, asthma/lung disease, rheumatic disease, skin disease, neurological disease, depression/mental illness, cancer and/or other chronic illness, problems following an accident or disability. These questions have been used previously [35,47]. These questions were used as a measure of disease burden which is a standard practice for studies that explore SRH [48].

SRH was assessed according to a five-point scale (very good, good, neither good nor poor, poor, very poor). In population studies, SRH is generally accepted by researchers as a valid measure to determine health status [49] with SRH also being a predictor of mortality and overall health [10,16].

### Methodological approach and research framework

The data was analyzed using three methods: a correlation matrix, binary logistic regressions and a structural equation modeling approach (SEM), for which a theoretical framework was constructed.

**Figure 1:**
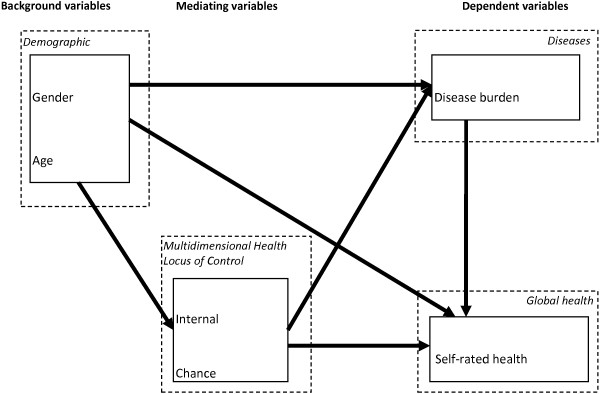
**Research framework and model construction.** This HDLoC model outlines how the theory was implemented in the empirical pathway analysis.

A research model of SRH influenced by disease burden and health locus of control factors (HDLoC) was constructed to examine the relationships between the variables (Figure 1). The model contains one dependent (SRH), four mediating (MHLC on three levels and disease burden), and three independent factors (gender, age and education). The research model was determined after logical reasoning considering the time factors of the variables and the previously known association presented in the introduction. MHLC is considered to be relatively stable over time [19,20], unlike SRH and disease burden that reflect a more present stage. The underlying assumption in the model is that people who score highly on the internal LoC scale (those who believe that their own health behavior determines their own health status) should be more likely to carry out healthier behaviors than someone who scores low on the same scale; this should by extension lead to higher SRH and less disease burden. In the same way if someone scores high on the LoC subscale regarding chance (thus believing that luck or chance determines their health status), they should be less likely to maintain healthy behavior and by extension have lower SRH and higher disease burden.

### Statistical analysis

Statistical Package for the Social Sciences (SPSS)® version 19 (Chicago, IL, USA) was used for descriptive statistics and the logistic regression and WarpPLS v. 3.0 was used for the structural equation modeling (SEM) analysis, with partial least squares estimation technique (PLS) [50]. SEM is a combination of confirmatory factor and path analysis, which allows for the inclusion of latent (not directly measured) variables [51]. Latent variables (LV) differ from observed sum-scores (index) of the indicators as they can account for measurement error in the items, and items are allowed differential weights when estimating the latent construct [52].

SEM was conducted using the PLS estimation technique with Wold’s algorithm [53-55]. SEM-PLS suits the purpose for this explorative study where the primary goal was to determine which factors influence SRH, disease burden and LoC, and how they relate to each other [56]. SEM-PLS has also been used for health behavior studies [57-59]. SEM works with two models: (I) a measurement model (also called the outer model) which determines the relationships between observed variables and their association to the LV; (II) a structural model (also called the inner model), relating LV to other LV. PLS estimates loadings and path parameters between LV and maximizes the variance explained for the dependent variables. SEM-PLS allows for the opportunity to determine direct, indirect and total effect of the independent variables in the model.

Model fit indicators in WarpPLS apply to the degree of association between the observed data and the model-implied data. In WarpPLS, the output model fit is assessed by three indices: Average path coefficient (APC), Average R-squared (ARS) and Average variance inflation factor (AVIF). APC and ARS should be under two and significant (P < 0.05). AVIF is recommended to be lower than five.

### Ethical consideration

Ethical approval was sought at the regional Ethical Committee of Clinical Investigation in Uppsala but was not deemed necessary according to Swedish law since the study group responded anonymously, leaving no possibility of individual identification.

## Results

The average age of the study population was 64 years and consisted of slightly more men than women. Compulsory school was the most common education level completed. The distribution of demographics and key variables in the study population are shown in Table 1.

**Table 1:** Characteristics of male and female participants

		**Male (51%)**	**Female (49%)**	**Total**
Age	Mean (SD)	63.5 (10.1)	64.9 (8.8)	64 (9.5)
Education	Compulsory school	36.0	44.1	40.0
	Secondary school	29.9	28.2	29.1
	University	34.1	27.7	31.0
MHLC^a^	Internal (MD)	24**	22**	23
	Chance (MD)	16	17	17
	Powerful others (MD)	20**	18**	19
Disease burden (number of diseases)	≤2	61.3	56.6	59.0
	3	21.6	21.2	21.4
	4	8.8	15.2	11.9
	≥5	8.3	7.1	7.7
Self-rated health	Very poor	0.5	0	0.2
	Poor	6.2	8.9	7.5
	Neither good nor poor	31.4	25.7	28.6
	Good	51.0	56.9	53.9
	Very good	11.0	8.4	7.7

Figures aspercentages if not stated otherwise.

^a^Multidimensional health locus of control in index-form and with median values (MD).

**P < 0.01.

### Multidimensional health locus of control

As can be observed in Table 1, there was a significant difference with internal and powerful others between males and females, while there was no significant difference with chance.

### Disease burden and SRH

Almost 60% of the group had two diseases or less, while 7.7% of the group had five diseases or more. There was no significant difference between males and females for disease burden.

A majority (61.6%) of patients reported good or very good global health and 7.7% reported poor or very poor global health. The last third reported neither good nor poor health. There was no significant difference between males and females for SRH.

### Correlation matrix and logistic regression

Several associations outlined in the research framework (Figure 1) were significant in the correlation matrix (Table 2). The highest correlations with SRH were seen with disease burden and internal MHLC.

**Table 2:** Correlation matrix among indicators

	**Min**	**Max**	**Std-Dev**	**1**	**2**	**3**	**4**	**5**	**6**	**7**
1. Gender	1	2	.50							
2. Age	22	89	9.52	.06						
3. Education level	1	3	.84	-.09	-.27**					
4. Internal MHLC^a^	6	36	5.37	-.16**	-.05	-.03				
5. Chance MHLC^a^	6	33	4.59	.02	.13**	-.28**	.17**			
6. Powerful others MHLC^a^	7	36	5.26	-.22**	.24**	-.11*	.22**	.22**		
7. Disease burden	0	9	1.60	.05	.06	-.02	-.13**	.14**	.02	
8. Self-rated health	1	5	.77	.00	-.09	.10*	.22**	-.18**	-.14**	-.36**

*Correlation is significant at the 0.05 level.

**Correlation is significant at the 0.01 level.

^a^Multidimensional health locus of control in index-form, ranging from 6 to 36.

Maximum (max), minimum (min), standard deviations (Std-Dev) and correlations indicated. The matrix has been calculated with Spearman’s correlation coefficient. An index from 6 to 36 was used for each dimension of health locus of control.

The background variables and LoC were tested using a logistic regression with SRH dichotomized into either good or less than good (Table 3). Logistic regression Model 1 tested the background variables’ associations to SRH while Model 2 tested the three dimensions of MHLC with SRH. In Model 2, all dimensions of LoC had significant relationships with SRH. These results remained consistent for internal and powerful others MHLC but not for chance and then the model was adjusted with the three background variables as well as disease burden (Model 3).

**Table 3:** Logistic regressions: Associations between background variables, locus of control, disease burden and self-rated health

	**Variables**	**Nagelkerke r**^**2**^	**B**	**S.E.**	**OR**	**95% CI**	**P-value**
Model 1		0.02					
Background	Gender^a^		.199	.207	1.220	0.81 to 1.83	0.337
	Age		-.015	.012	.985	0.96 to 1.01	0.199
	Education level		.151	.129	1.163	0.90 to 1.50	0.243
Model 2		0.09					
MHLC^b^	Internal		.082	.023	1.085	1.04 to 1.14	0.000
	Chance		-.061	.026	.940	0.89 to 0.99	0.017
	Powerful others		-.066	.023	.936	0.90 to 0.98	0.004
Model 3		0.19					
Background	Gender^a^		.243	.245	1.275	0.79 to 2.06	0.321
	Age		-.007	.014	.993	0.97 to 1.02	0.625
	Education level		.182	.151	1.200	0.89 to 1.61	0.230
MHLC^b^	Internal		.063	.025	1.065	1.01 to 1.12	0.012
	Chance		-.038	.029	.963	0.91 to 1.02	0.188
	Powerful others		-.056	.025	.945	0.90 to 1.00	0.026
Diseases	Disease burden		-.416	.080	.660	0.56 to 0.77	0.000

^a^Men = 1, women = 2.

^b^Multidimensional health locus of control in index-form, ranging from 6 to 36.

Odds ratio (OR), standard error (S.E.) and confidence interval (CI) for the logistic regressions.

### SEM analyses of MHLC, disease burden and SRH

**Figure 2:**
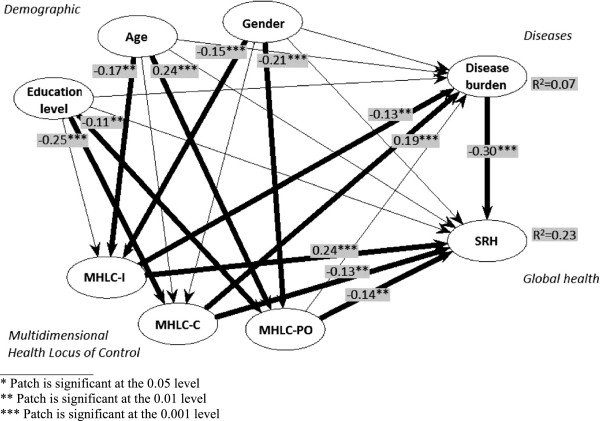
**Structural equation model analysis of data outlined after the theoretical framework generated through a partial least squares estimation technique, with path coefficients of the pathway model (i.e. inner model). **The model outlines the hypothesized relationships among the factors in the HDLoC model. Significant direct associations between latent variables are presented in bold.

The SEM analyses of MHLC, disease burden, SRH and the demographic variables (Figure 2) showed that the three background variables were directly associated with several dimensions of LoC, but not with SRH or disease burden (Table 4).

**Table 4:** Path coefficients and p-values of direct effects on LoC, disease burden and self-rated health

**Background variables**	**Mediating variables**								**Dependent variable**	
	**MHLC-I**^**a**^		**MHLC-C**^**a**^		**MHLC-PO**^**a**^		**Disease burden**		**Self-rated health**	
	**PC**	**p-value**	**PC**	**p-value**	**PC**	**p-value**	**PC**	**p-value**	**PC**	**p-value**
Gender	-0.15	<0.001	-0.04	N.s.^b^	-0.209	<0.001	0.04	N.s.^b^	0.02	N.s.^b^
Age	-0.17	0.004	0.12	0.010	0.24	<0.001	0.08	N.s.^b^	-0.05	N.s.^b^
Education level	-0.05	N.s.^b^	-0.25	<0.001	-0.109	0.008	0.02	N.s.^b^	0.03	N.s.^b^
MHLC-I^a^							-0.13	0.002	0.24	<0.001
MHLC-C^a^							0.19	<0.001	-0.14	0.002
MHLC-PO^a^							0.06	N.s.^b^	-0.14	0.002
Disease burden									-0.30	<0.001

^a^Multidimensional health locus of control in intern (-I), chance (-C) and powerful other (-PO).

^b^Not significant.

Path coefficients (PC) for direct effects in the structural equation model.

High internal MHLC was positively associated with SRH, while high MHLC in chance and powerful others were negatively associated with SRH. Disease burden was negatively associated with internal MHLC and positively associated to chance MHLC. Disease burden also seems to lower SRH, a result that was further supported in the logistic regression (Table 3).

The total model fit indices were good with APC = 0.124 (P < 0.001), ARS = 0.112 (P < 0.001) and AVIF = 1.074.

### Indirect and total effects in the SEM analyses

None of the demographic variables were directly associated with disease burden or SRH in the path model (Figure 2). However, age and education level had indirect significant associations (Table 5). Age was negatively associated with SRH and positively associated with disease burden. Having higher education was associated with having better SRH and less disease burden.

**Table 5:** Indirect and total effects on disease burden and self-rated health

	**Disease burden**				**Self-rated health**			
	**Indirect effects**		**Total effects**		**Indirect effects**		**Total effects**	
	**PC**	**p-value**	**PC.**	**p-value**	**PC.**	**p-value**	**PC.**	**p-value**
Gender	0.01	N.s.^b^	0.05	N.s.^b^	-0.02	N.s.^b^	0.00	N.s.^b^
Age	0.06	0.026	0.14	N.s.^b^	-0.13	0.002	-0.18	N.s.^b^
Education level	-0.05	0.012	-0.03	N.s.^b^	0.04	0.034	0.07	N.s.^b^
MHLC-I^a^					0.04	0.010	0.28	<0.001
MHLC-C^a^					-0.06	<0.001	-0.19	<0.001
MHLC-PO^a^					-0.02	N.s.^b^	-0.16	0.001

^a^Multidimensional health locus of control in intern (-I), chance (-C) and powerful other (-PO).

^b^Not significant.

Path coefficients (PC) for indirect and total effects in the structural equation model.

## Discussion

This study aimed to explore how health LoC and disease burden are associated with SRH. Additionally, this study tested a model, HDLoC, that could contribute to the understanding and predictability of SRH, as well as SRH’s association to LoC and disease burden.

Most people in this study rated their health as good or very good. Health locus of control and disease burden were strongly associated with SRH. One interesting finding was the positive association between internal MHLC and SRH, and the negative association between internal MHLC and disease burden. This indicates that a high internal MHLC may have a direct impact on perceived health and an additional effect through disease burden, while disease burden has an association with SRH. This suggests that internal MHLC had both high indirect and total associations with SRH (Table 4). These results are consistent with other longitudinal studies on LoC [23]. Previous studies have also shown that internal health locus of control have associations with healthier choices and healthier behaviors [60], and cohort studies have shown that high internal LoC seems to be associated with a reduced risk for common chronic diseases such as CVD and cancer [27]. These results may explain some of the associations in our results. MHLC in chance seems to have the opposite relationship with health and diseases, as it lowers SRH and enlarges disease burden. High MHLC in powerful others had a negative association to SRH. Disease burden was also shown to mediate effects from both MHLC and background characteristics with SRH. This is consistent with Grotz et al.’s findings that high LoC in chance can be regarded as a risk factor for unhealthy behavior [61]. However the mechanism between LoC and SRH is not indisputable and the effect is not likely to be a non-complex function of healthy behavior.

Age and education level had no direct, but indirect, associations with disease burden and SRH. This indirect association was through MHLC, suggesting that MHLC mediates the effect of age and education. Earlier studies have shown that LoC has varied with age [62] with those older acknowledging the importance of external sources of control and at the same time preserving their sense of internal control [63]. Our study found that higher education has negative associations with chance and powerful others MHLC. Previous studies have also found that a high level of education is associated with lower scores on the external scales [64]. Higher education seems to lower disease burden and increase SRH through MHLC. Age has the opposite association. These results are only apparent in the SEM, and not in the logistic regression, which only measures direct effects. From a methodological point of view, these results indicate that it is important to measure indirect effects in order to see how the variables are associated with each other in a model.The HDLoC model (Figure 1) was created to test associations of demographics and health locus of control on disease burden and SRH. It can be discussed whether the directions or causality arrows in the model are appropriate. This was a cross-sectional study and therefore the directions were based on logical reasoning and the chronology with which these factors are assumed to have.

The variables outlined in the HDLoC model could explain 23% of the variance in SRH, which leaves 77% unexplained variance in SRH that is dependent on factors which were not assessed in this model. This indicates that there are other variables which impact SRH. There are other variables which have an impact on SRH and it may not be unexpected that SRH is perhaps the most inclusive measure of health reflecting measures available to date, and for this reason SRH has a high predictive capacity for survival which are not covered by other health indicators [29]. However, the SEM model could explain more of the variance in SRH than the logistic regression model.

In order to test a population with chronic disease, patients visiting a pharmacy to receive their statin treatment were used as the sample population. This resulted in a high response rate and a sample population with a relatively high age. Patients with poor adherence to statins are likely to be underrepresented in this study group. Report bias may exist due to the nature of self-reported data.

This study suggests that it may be possible to increase an individual’s perceived health and lower disease burden by managing LoC and factors that are related to LoC. Although LoC is quite stable over time and not easy to change, targeted interventions have demonstrated changes in LoC [21]. More research is needed to determine if these types of changes are stable or if health LoC returns to its original level over time. This study also highlights the importance of disease burden for perceived health, and that LoC factors probably are important for both perceived health and the development of diseases, which also seems to be important for perceived health. The results of this study suggest that an approach targeting LoC might be able to increase SRH. However LoC is not assumed to be easily affected as it is considered to be partly a trait-like and partly a state-like measure [35]. LoC is supposed to be similar in a variety of health-related situations, but may also be sensitive enough to change as a function of one’s health-related experiences. In which case it will probably take a long time before a change in LoC can cause a change in SRH, as there are many mechanisms, healthy choices and behaviors that must be affected for a change to be noticeable in SRH or in disease burden. The implications of this study are primarily on a theoretical level, as more research is needed on the mechanisms underlying SRH and LoC. However this study suggests that LoC should be considered an important factor included in studies of personal perceived health, and might be beneficial to work with LoC factors in patient groups with long-term diseases.

## Conclusion

This study suggests that SRH is positively associated with internal health locus of control and negatively associated with chance and powerful others health locus of control, as well as disease burden. Disease burden is negatively associated with internal health locus of control but positively associated with chance health locus of control.

## Abbreviations

APC: Average path coefficient; ARS: Average R-squared; AVIF: Average variance inflation factor; CVD: Cardiovascular disease; HDLoC: Self-rated Health influenced by Disease burden and health Locus of Control factors; LoC: Locus of Control; LV: Latent variables; MHLC: Multidimensional Health Locus of Control; PLS: Partial least squares; SEM: Structural equation modeling; SRH: Self-rated health; SPSS: Statistical Package for the Social Sciences.

## Competing interests

The authors declare that they have no competing interests.

## Authors’ contributions

EB designed the study, undertook the statistical modeling and led the writing. PL and RW designed the study, contributed to data interpretation, and commented on successive drafts of the manuscript and handling of data. All authors approved the final version of the manuscript.

## Pre-publication history

The pre-publication history for this paper can be accessed here:

http://www.biomedcentral.com/1471-2458/14/492/prepub
